# Relationship between freshwater harmful algal blooms and neurodegenerative disease incidence rates in South Korea

**DOI:** 10.1186/s12940-022-00935-y

**Published:** 2022-11-26

**Authors:** Seungjun Lee, Boseung Choi, Sung Jae Kim, Jinnam Kim, Dayun Kang, Jiyoung Lee

**Affiliations:** 1 Department of Food Science and Nutrition, College of Fisheries Science, Pukyong National University, Busan, Republic of Korea; 2 Division of Big Data Science, Korea University, Sejong, Republic of Korea; 3 Biomedical Mathematics Group, Institute for Basic Science, Daejeon, Republic of Korea; 4 Department of Economics and Statistics, Korea University, Sejong, Republic of Korea; 5 Department of Biology, Kyungsung University, Busan, Republic of Korea; 6 College of Public Health, Division of Environmental Health Sciences, The Ohio State University, 406 Cunz Hall, 1841 Neil Avenue, Columbus, OH 43210 USA; 7 Department of Food Science and Technology, The Ohio State University, Columbus, OH USA; 8 Infectious Diseases Institute, The Ohio State University, Columbus, OH USA

**Keywords:** Alzheimer’s disease, Parkinson’s disease, Gridded population, BMAA, Microcystin, Aerosol

## Abstract

**Background:**

Due to anthropogenic activities and global warming, the severity and distribution of harmful algal blooms (HABs) have been increasing steadily worldwide, including in South Korea (S. Korea). Previous studies reported that exposure to HABs could increase the risk of HAB-related diseases. However, very few studies examined the linkage between HABs and disease occurrence, particularly in S. Korea. The objective of this study was to evaluate the potential impact of HABs on neurodegenerative diseases (NDs), including Alzheimer’s disease, Parkinson’s disease, and motor neuron disease, at a population level.

**Methods:**

Thirteen-year data (2005–2017) for chlorophyll-*a* (chl-a) concentrations as a bloom-related parameter, annual numbers of NDs, and population information were collected. First, the entire area of S. Korea was divided into a grid of 1 km, and the population number in each 1-km grid was collected using the Statistical Geographic Information Service Plus system. Cross-sectional time series data were analyzed with two statistical models, a generalized linear mixed model and a generalized linear model.

**Results:**

The results show a general trend of increasing chl-a concentration and NDs year by year. We observed positive correlations between HAB intensity and the incidence rate of NDs. Particularly, HABs seem to have the most long-term carry-over effect on Parkinson’s disease. Another key finding was that a 5-km radius from the HAB location was the boundary that showed the most significant associations with three NDs.

**Conclusions:**

This study provides statistical evidence that supports the potential risk of NDs from the exposure to HAB. Thus, it is recommended to monitor a broad spectrum of cyanotoxins, including neurotoxins, in bloom-affected regions in S. Korea and epidemiological studies in the future.

**Supplementary Information:**

The online version contains supplementary material available at 10.1186/s12940-022-00935-y.

## Background

Cyanobacterial harmful algal blooms (HABs) in freshwater have been intensified by eutrophication and climate change and expanded globally [1]. A significant public health concern of HABs is that some species of cyanobacteria produce toxic metabolites, known as cyanotoxins, such as microcystin, nodularin, cylindrospermopsin, anatoxin-a, and saxitoxins, so threaten water bodies used as water resources. Cyanotoxins have neural, dermal, gastrointestinal, or/and hepatic toxicities [2, 3]. Previous studies summarized that cyanotoxin exposure pathways include ingestion, inhalation, and dermal contact, and those exposures have links to animal and human health risks [4]. A handful of studies reported HAB exposure and related health risks at a population level. Zhang et al. [5] and Lee et al. [2] reported a significant positive association between cyanobacterial blooms and non-alcoholic liver disease in the United States (US) and South Korea, respectively. In addition, Li et al. [6] revealed the relationship between chronic microcystin exposure and liver damage in children in China. Gorham et al. [7] showed that using cyanobacterial bloom-affected water for a drinking water source is a significant risk factor for increased hepatocellular carcinoma incidence rates in Ohio, US and Svirčev et al. [8] reported a possible connection between HABs and multiple cancers, including brain cancer and primary liver cancer, in Serbia.

Compared to hepatotoxic effects, incidences of cyanobacterial neurotoxin poisoning are less frequently documented [1]. Certain cyanobacteria species produce neurotoxic metabolites in freshwater. Saxitoxins, anatoxin-a, and paralytic shellfish toxins are the major neurotoxins, and β-N-methylamino-L-alanine (BMAA) is the bioactive peptide that is the most commonly reported as a risk factor for amyotrophic lateral sclerosis (ALS) [9, 10]. BMAA was detected in the brain tissue of Alzheimer’s disease (AD) patients in the US and Canada [11]. Humans can be exposed to these neurotoxins via various routes, such as drinking water, recreational water activities, aerosols, and food [1, 12]. Previous studies reported BMAA accumulation in fish and shellfish [13–15] which can be an exposure pathway to higher organisms. Some reports concluded that the causal relationship between BMAA and ALS/PDC and BMAA is not supported by existing data [16]. However, there has been accumulating evidence that long-term exposure to cyanotoxins is being identified as having neurotoxic effects [1]. BMAA is prevalent in aquatic environments [17] and in the air (Supplementary information Fig. 1 and Table 2). BMAA also exists in terrestrial environments and one example is agricultural fields where water treatment sludge is disposed of in bloom-affected areas [18]. Thus, there is a possibility that humans can be exposed to neurotoxins in various exposure pathways that have not been explored or imagined before, and there is an imperative need to study the impact of HABs on neurodegenerative diseases in the areas affected by HABs around the world.

The main goal of this study was to determine whether there are significant associations between HABs and neurodegenerative diseases (NDs) at a population level. For this, the current study analyzed three NDs (motor neuron disease (MND), AD, and Parkinson’s disease (PD)), incidence rates in a 1-km grid population in the Republic of Korea (hereafter South Korea (S. Korea)) from 2005 to 2017. In S. Korea, four major rivers are critically important for water resources for the nation, but those rivers have been infested with severe blooms for recent decades [2]. However, reports of potential impacts of HAB on human health are extremely limited. To our knowledge, this is the first study to investigate a possible linkage between HABs and ND rates at a human population level. Key findings support statistical evidence between HAB severity and neurodegenerative disease risks, so we recommend future studies for comprehensive monitoring of various exposure pathways and determining a causal relationship between HAB severity and human neurodegenerative diseases.

## Methods

### Collection of neurodegenerative diseases and bloom data

To identify the association between harmful algal blooms and NDs, chlorophyll-a (chl-a) concentration data and the annual number of NDs for an administrative unit (‘gu’ or ‘gun’) in S. Korea were used. Since the observed sites where chl-a concentrations were measured were located along the rivers and lakes, the administrative units were not accurately matched geographically. Thus, a gridded population was used to solve this problem, which are estimate of the people in a grid cell, derived with a geo-statistical model using census or small area population counts and several other spatial datasets [19].

For bloom data, chl-a concentrations from 939 locations were obtained from the Water Information System at the Korean Ministry of the Environment. Since monitoring of cyanotoxins was limited and has not been conducted regularly, chl-a concentration was used as a bloom intensity indicator; the chl-a data are the only available bloom parameter covering the entire areas along the rivers and lakes during the study period from 2005 to 2017. Annual average chl-a concentrations per location were used for analysis. A Korean governmental agency conducts monitoring of cyanobacterial cell counts, geosmin, and chl-a concentrations during HAB seasons. Measurements of BMAA, anatoxins, or saxitoxin are rarely performed, and data are not available. Therefore, in this study, chl-a concentration was used as a bloom indicator.

### Conversion to gridded population

In order to map the annual HAB and ND data, the longitude and latitude information was collected for all 939 chl-a monitoring locations. Then the data were converted into geographical information system using QGIS software (version 3.22.2, https://qgis.org/en/site/). The entire area of S. Korea was divided into a grid of 1 km, and then 127,595 points of the grid were generated. Next, the 939 observed locations were mapped onto the entire grid points. If two or more observation locations of chl-a correspond to one grid, the median value of chl-a concentration was used and then mapped onto the grid.

**Fig. 1:**
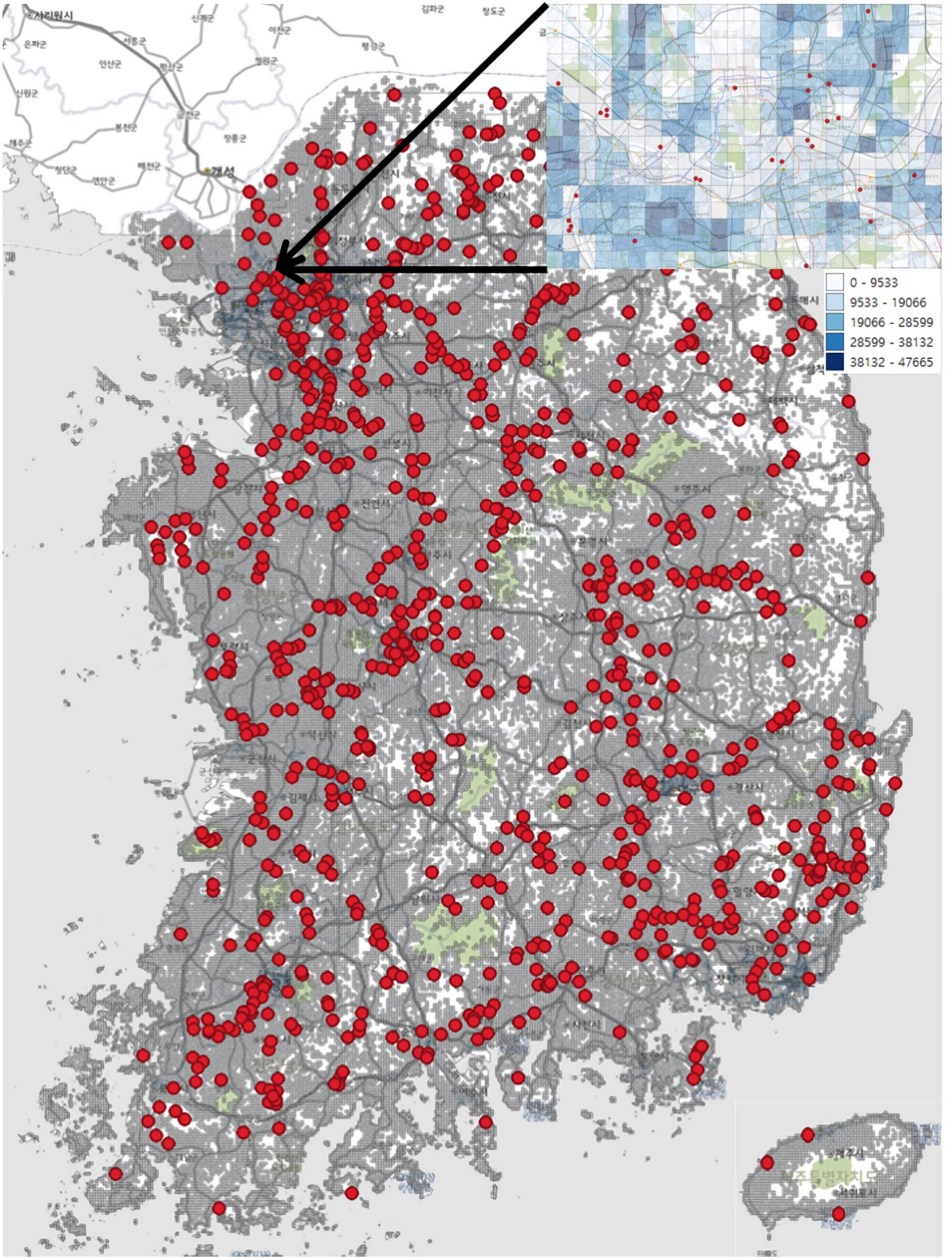
The locations of chlorophyll-*a* monitored sites (red dots) and 1-km grids (grey lines) for extracting population information in S. Korea. For example, the upper right window shows zoomed-in information on the Seoul area (darker blue represents a higher population number)

Next, the population number in each 1-km grid was collected using the SGIS (Statistical Geographic Information Service) Plus (https://sgis.kostat.go.kr) system. SGIS Plus is a location-based open service platform linked and fused with public and private data and census data of population, households, houses, and businesses owned by Statistics Korea (Korean Bureau of Census). SGIS Plus provides a variety of statistical data according to the size of the grids. The smallest size of the grid is 100 m, and the largest is 100 km. The total population and numbers of over 65 years old were extracted for every 1-km grid from the entire areas of S. Korea. In Fig. 1, red dots represent all observed locations of chl-a concentration, and grey lines are 1-km grids. For the surrounding areas in Seoul, the capital, the 1-km grids are not distinguishable due to its highly dense population. Thus, it was enlarged for displaying better granularity.

**Fig. 2:**
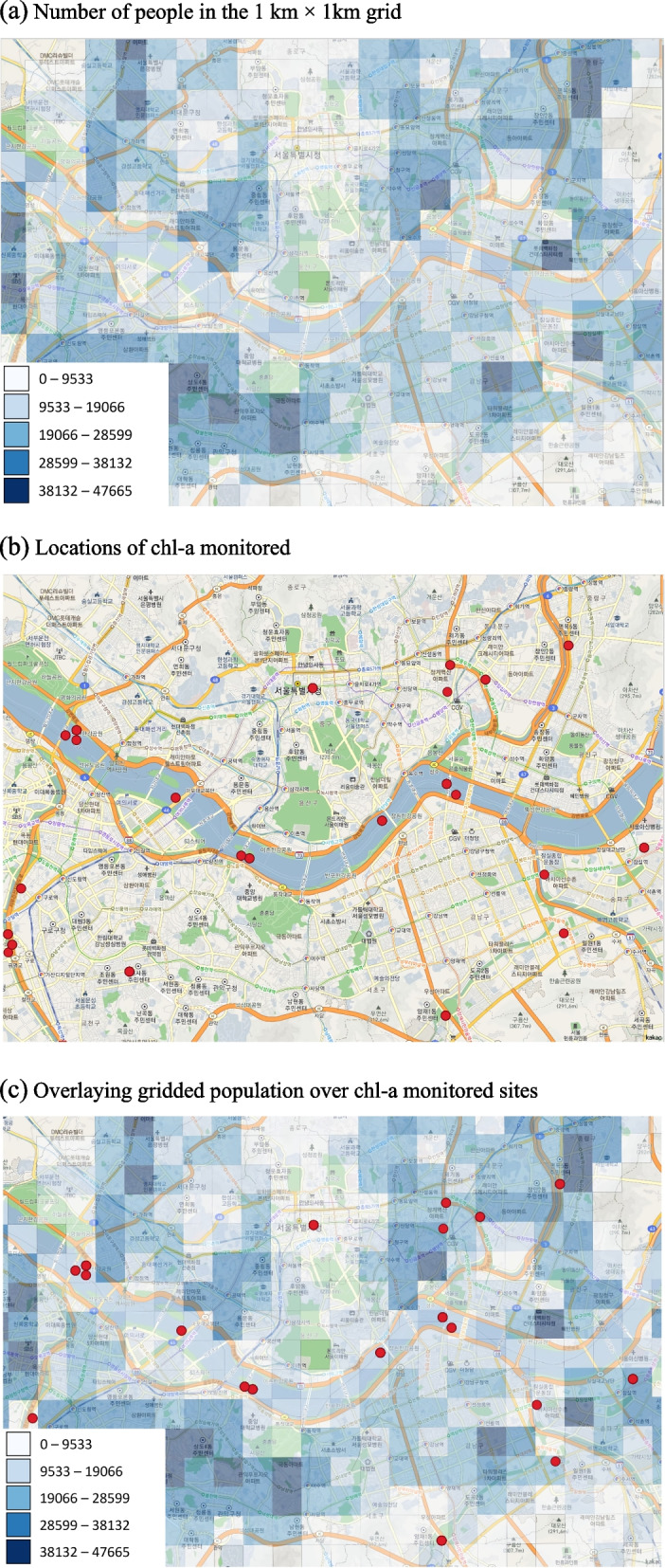
The approach used for digitization of chlorophyll-*a* monitored sites. Examples are shown using the sites in Seoul with a large river transecting the city and many tributary streams. (**a**) Number of people in the 1 km × 1 km grid. (**b**) Locations of chl-a monitored. (**c**) Overlaying gridded population over chl-a monitored sites

Figure 2 depicts more detailed information on the Seoul area. In Fig. 2, panel (a) illustrates the number of people in the 1-km grid in Seoul. The darker the color is, the larger the number of populations in the area is. Panel (b) represents the observed location of the chl-a concentration measured. It shows that chl-a concentrations were monitored along the rivers and streams. Panel (c) overlays the gridded population and chl-a concentration observed locations. A benefit of using a 1-km gridded population is its scalability: we can easily extend the grid size by aggregating the grids into 3-km or 5-km grids and then matching each extended grid with observed chl-a concentration data.

### Cross-sectional time series data

After collecting chl-a concentration data over 13 years (from 2005 to 2017), we matched the gridded population to these observed years. However, SGIS Plus does not provide data every year. Statistics Korea conducted the Census every five years until 2015. Therefore, gridded population data in 2005, 2010, and 2015 were used. In 2015, Statistics Korea performed a register-based Census, and census data have been generated yearly since then. Thus, based on the Censuses, we could have the gridded population data in 2005, 2010, 2015, 2016, and 2017. To match chl-a concentration data for those 13 years to the irregularly observed gridded population, we had to expand the population data to 13 years. Thus, we used an interpolation method of missing values in time series by connecting consecutive non-missing input values with the spline method [20]. Finally, we had population estimates for these missing years of 2006, 2007, 2008, 2009, 2011, 2012, 2013, and 2014.

In this study, three NDs were considered; MND, AD, and PD. The ICD-10-CM diagnosis codes were G12.2 for MND, G30.0 for AD, and G20 for PD. In addition, the codes of three diseases were converted from the ICD-9 codes (before 2015 data) to the ICD-10 codes. The annual numbers of patients with the three diseases from the National Health Insurance Service were collected, and the data were rearranged according to the administrative units of the district. In S. Korea, a unit of district-level has three types: “Si” as a city, “Gu” as a district in metropolitan cities only, and “Gun” as a division within provinces. The total number of administrative units is 226, less than the total number of the 1-km grid and less than the number of chl-a observed locations (939 locations). Due to the lack of exact congruency between 1-km grid, chl-a marked locations, and the of administrative units, we had to match the patient number to the corresponding chl-a concentration location using the below method.

**Fig. 3:**
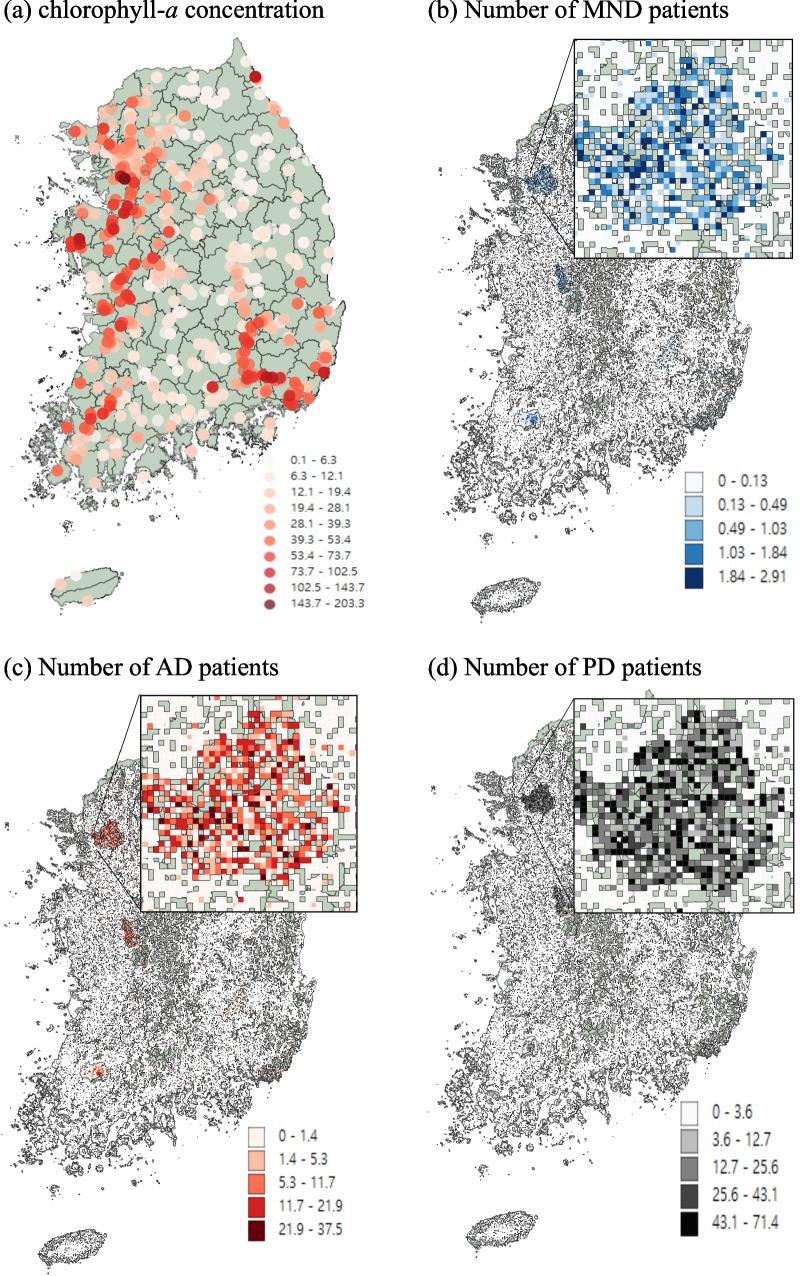
Heatmaps of average concentrations of chlorophyll-*a* (**a**), the average adjusted numbers of MND patients (**b**), AD patients (**c**), and PD patients (**d**) in a 1-km grid. Data are visualized here using the last three years of the study period (2015–2017)

The number of patients for each administrative district in the 1-km grid was evenly distributed according to the populations of each grid. Let *x*_*it*_ be the number of senior patients in the *i*^*th*^ administrative unit at time *t* and *y*_*jt*_ be the number of ND patients in the *j*^*th*^ 1- km grid at the same time *t*. The modified number of patient *y*_*jt*_ is given by in Eq. (1).

1 $${\boldsymbol{y}}_{\boldsymbol{jt}}={\boldsymbol{x}}_{\boldsymbol{it}}\times \frac{{\boldsymbol{G}}_{\boldsymbol{jt}}}{{\boldsymbol{P}}_{\boldsymbol{it}}},\boldsymbol{i}=\textbf{1},\textbf{2},\dots, \textbf{226},\boldsymbol{j}=\textbf{1},\textbf{2},\dots, {\boldsymbol{n}}_{\boldsymbol{i}},\boldsymbol{t}=\textbf{2005},\dots, \textbf{2017}$$

where, *P*_*it*_ is the number of total populations in the *i*^*th*^ administrative unit at time *t*, and *G*_*jt*_ is the number of people in the *j*^*th*^ 1- km grid, which is one of the administrative units at time *t*. Figure 3 depicts the heatmaps of the modified numbers of patients in a 1-km grid. From panels (b) to (d), each panel represents the heatmap of the modified number of ND patients of MND, AD, and PD, respectively. Since much of the population live in the Seoul metropolitan area, about one-quarter of the people of S. Korea, we zoomed in on the area. The zoom-in area can be seen in the upper right corner of each panel. Other dark-colored areas are another regional capital of S. Korea. The upper left panel (a) represents the concentration of the observed spot of chl-*a*. We can see that the higher concentrations are located following the four big rivers in S. Korea.

As NDs are related to old people, the number of patients in the 1-km grid needs to be adjusted after considering the population size of older people (> 65 years old). So we performed the second modification of the number of diseases. Eq. (2) represents the second modified number of NDs according to the more aging population.

2 $${\boldsymbol{y}}_{\boldsymbol{jt}}={\boldsymbol{x}}_{\boldsymbol{it}}\times \frac{\textbf{1}}{\boldsymbol{P}{\textbf{65}}_{\boldsymbol{it}}}\times \frac{{\boldsymbol{G}}_{\boldsymbol{jt}}}{{\boldsymbol{P}}_{\boldsymbol{it}}},\boldsymbol{i}=\textbf{1},\textbf{2},\dots, \textbf{226},\boldsymbol{j}=\textbf{1},\textbf{2},\dots, {\boldsymbol{n}}_{\boldsymbol{i}},\boldsymbol{t}=\textbf{2005},\dots, \textbf{2017}$$

where *P*65_*it*_ is the number of populations over 65 in the *i*^*th*^ administrative unit at time *t*, the other variables are the same with Eq. (1). The first product on the right side of Eq. (2) is the corrected number of ND patients according to the number of the population over 65 years in the administrative unit. The updated number was revised by multiplying the 1-km grid population and administrative unit population ratio. The final corrected number of patients was calculated for every three ND and was used as a response variable in the statistical model.

### Statistical analysis

In order to examine the relationships between the HABs and the numbers of three NDs, we had to figure out two issues first: 1) to evaluate the long-lasting effects of HABs on the NDs, and 2) to confirm the spatial distance impact of HABs on the three NDs. We applied generalized linear mixed models (GLMMs) for the first issue and used generalized linear models (GLMs) for the second one. For the GLMMs, we set the log link function and the normal distribution for a random component. In addition, to identify a serial correlation that the repeated measured response variable might have, we added an one-way random effect to the model [21]. The main effects of HABs on the variation of NDs were considered fixed effects in the model. Let ${y}_{jt}^k$ be the corrected number of *k*^th^ ND defined in Eq. (2), where *k* = 1, 2, 3, 1 is for MNDs, 2 for ADs, and 3 for PDs and *z*_*jt*_ be the observed concentration of chl-a with the same time point and location. We modeled the number of NDs for the effects of HABs in the following:

3 $$\textbf{log}\left({\boldsymbol{y}}_{\boldsymbol{jt}}^{\boldsymbol{k}}\right)={\boldsymbol{\beta}}_{\textbf{0}}+\sum_{\boldsymbol{l}=\textbf{0}}^{\boldsymbol{\infty}}{\boldsymbol{\beta}}_{\boldsymbol{l}+\textbf{1}}{\boldsymbol{z}}_{\boldsymbol{j},\boldsymbol{t}-\boldsymbol{l}}+{\boldsymbol{u}}_{\boldsymbol{j}}+{\boldsymbol{\epsilon}}_{\boldsymbol{j}\boldsymbol{t}},\boldsymbol{k}=\textbf{1},\textbf{2},\textbf{3},\boldsymbol{j}=\textbf{1},\textbf{2},\dots, \textbf{939},\boldsymbol{t}=\textbf{2005},\textbf{2006},\dots, \textbf{2017}$$

where *u*_*j*_ is a random effect of *j*^*th*^ location, and we assumed that all *u*_*j*_ have zero mean and the same variance ${\sigma}_u^2$ and each *u*_*j*_ s is independent of each other, and *ϵ*_*jt*_ is an independently and normally distributed white noise. We could reflect the serial correlation within a location by including a random effect in the model. We performed the Hausman test to determine whether the model had to include a random effect [22]. The null hypothesis of the trial was that there is zero correlation between the fixed effects and random effects. If we did not reject the null hypothesis, the random effect model was better than the fixed effect model. Based on the results of the Hausman test, all three ND models showed that the null hypothesis did not reject all. Therefore, we could say that all three models have random effects.

For the second issue, we only utilized the 2017 data set because it is the most recent one among the complete data sets, had very few missing observations, and it may best reflect the spatial effect of HABs in the model compared to other data sets. To model the ND number response variable, GLMs were used:

4 $$\textbf{log}\left({\boldsymbol{y}}_{\boldsymbol{jr}}^{\boldsymbol{k}}\right)={\boldsymbol{\beta}}_{\textbf{0}}+{\boldsymbol{\beta}}_{\boldsymbol{jr}}{\boldsymbol{z}}_{\boldsymbol{j}}+{\boldsymbol{\epsilon}}_{\boldsymbol{j}},\boldsymbol{k}=\textbf{1},\textbf{2},\textbf{3},\boldsymbol{j}=\textbf{1},\textbf{2},\dots, \textbf{939}.$$

In this model, the subscript *k* and *j* are the same with Eq. (3). The subscript *r* represents the size of the radius. We examined the change of the effects of HABs on three ND response variables according to the radius increment by 3 km and 5 km (see Supplementary Information). When we used a 1-km grid for this purpose, many grids had no population as the locations for measuring chl-*a* concentrations were generally on the rivers if the width of the water bodies was bigger than 1 km and very close to the rivers, lakes, and streams. Thus, few people reside within those 1-km grids, and those grids with red dots on the river generate a lighter blue color due to the number of lower populations (Fig. 2(c)). Therefore, we used the primary grid sizes of 3 km and 5 km, instead of 1 km, for further statistical analyses.

## Results

**Fig. 4:**
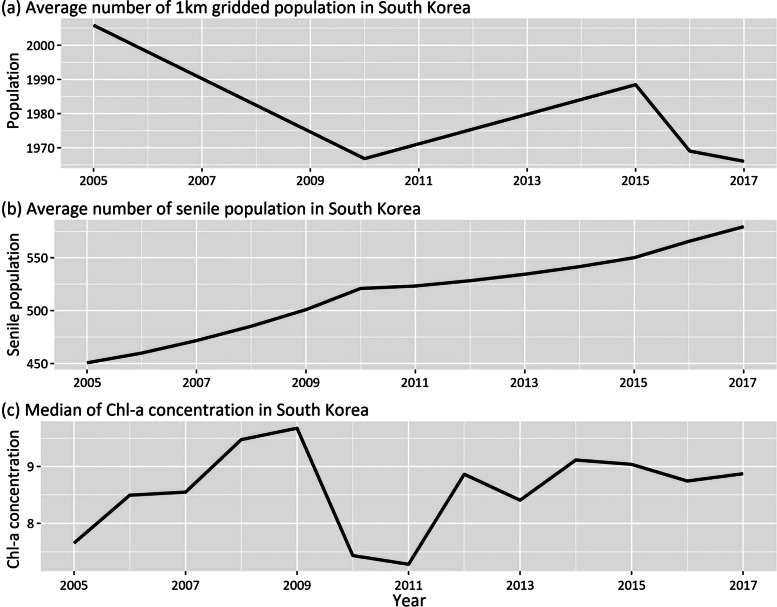
Trends of 1-km gridded population over time (**a**), numbers of the senior population older than 65 (**b**), and observed average chlorophyll-*a* concentrations (**c**) from 2005 to 2017 in S. Korea. (**a**) Mean number of 1-km gridded population. (**b**) Mean number of senior populations. (**c**) Median of chl-*a* concentrations

During the 13-year study period, both total and senior (> 65-year old) populations in 1-km grids increased, but the aging population increased faster than the general population (Fig. 4(a) and (b)). The chl-*a* concentration trend fluctuated, but it was generally on the rise (Fig. 4(c)).

**Fig. 5:**
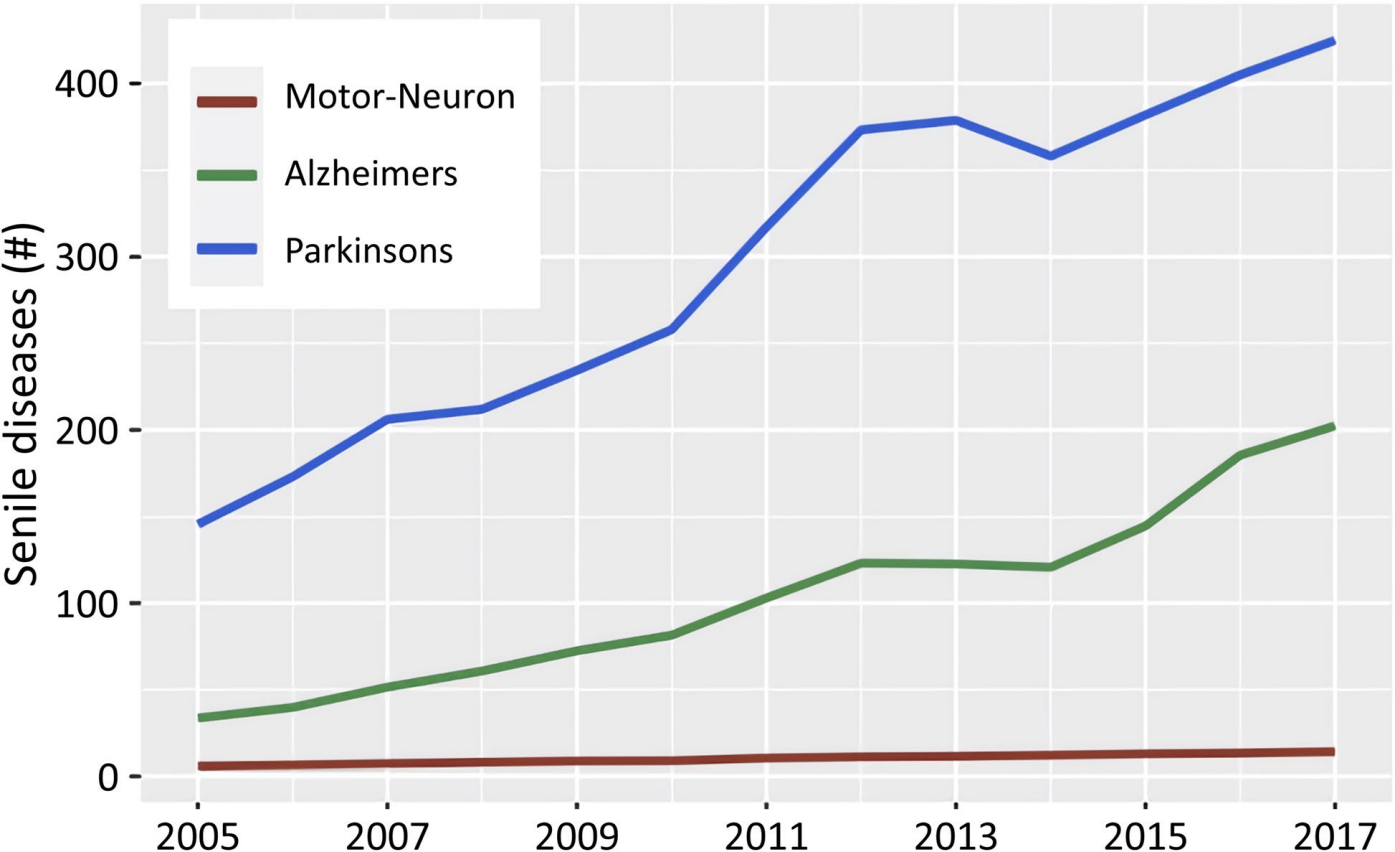
Comparison of the numbers of three types of neurodegenerative diseases in a 1-km gridded population from 2005 to 2017 in S. Korea (Blue: Parkinson’s disease; Green: Alzheimer’s disease; and Brown: motor neuron disease)

The trends of the three NDs are all increasing, but the varying degrees were different (Fig. 5). The increment of AD and PD numbers was much higher than the total number of MND during the study period, and the increasing speed of AD numbers was the most strikingly high (> four times increase) during the 13 years.

Table 1 shows the associations between HAB intensity and incidence rate of three NDs over time (e.g., MND, AD, and PD) and summarizes the estimation result of GLMMs with statistically significant coefficients. HAB_st_ represents the effect of HAB_s_ in the same year. HAB_st-1_ and HAB_st-2_ represent the effect of HABs 1 year and 2 years ago, respectively. In the case of MND, HABs measured in the same year were significantly correlated with the MND incidence rate. In contrast, the AD incidence rate was affected by HAB intensity measured 1 year ago and that of the same year. HABs continue to affect the PD incidence rate for the past 3 years up to the present. This result implies that HABs had the most long-term carry-over effect on PD, which gradually diminished as time passed. Among the three NDs, HABs seemed to have the most long-lasting impact on PD, while MND is relatively the least affected.

**Table 1:** Results of one-way random effect model that examined the relationship between HABs and neurodegenerative diseases ^a^

	Variable	Estimate	Standard Error	t	*P*
MND	Intercept	−11.9509	0.1051	− 113.74	<.0001
	HABs_t_	0.045745	0.0129	3.55	0.0004
Alzheimer’s disease	Intercept	−9.87655	0.961	− 102.77	<.0001
	HABs_t_	0.104222	0.0182	5.73	<.0001
	HABs_t-1_	0.071398	0.0181	3.94	<.0001
Parkinson’s disease	Intercept	−8.53985	0.0931	− 91.74	<.0001
	HABs_t_	0.069811	0.0115	6.06	<.0001
	HABs_t-1_	0.045031	0.0116	3.89	0.0001
	HABs_t-2_	0.040915	0.0114	3.59	0.0003
	HABs_t-3_	0.034051	0.0109	3.12	0.0018

*MND* Motor neuron disease

^a^Based on the numbers of diseases in each grid’s adjusted population as defined in Eq. (2)

Next, this study evaluated the effects of HABs on the incidence rate of the three diseases (MND, AD, PD) at different geographic distances (3 km and 5 km). Two radii based on the measurement point of chl-a concentration were applied (3 km and 5 km). The predictor is the same for each disease response model, and chl-a concentration and the response variables are the numbers of diseases for two different areas. Supplementary Table 1 summarizes the estimation result of GLMs for the model Eq. (4) and their significance. Similar patterns of statistical significance among all three diseases were observed: incidence rates at a 5 km radius showed more significant associations with the HABs than at 3 km.

## Discussion

Freshwater HABs are a serious risk affecting environmental and public health worldwide, especially due to their ability to produce various cyanotoxins [4, 23]. Our previous study demonstrated that the distribution and severity of HAB events have increased near the major rivers for the past decades in S. Korea [2]. Moreover, *Microcystis*, *Anabaena,* and *Oscillatoria* were known to be predominant in S. Korea from spring to autumn, producing anatoxins, saxitoxins, and BMAA [2, 24]. Kim et al. [25] reported saxitioxin producers measured by *sxtA* and *sxtG* genes in Han River, S. Korea. In addition, anatoxin-a was detected in several lakes (e.g. Chungju Lake, Jangsong Lake, Youngsan Lake, and Younglang Lake), ranging from 417 to 1444 μg/g of freeze-dried bloom materials.

Previous studies reported that exposure to HABs could increase potential risks to human health, such as non-alcoholic liver diseases and ALS [2, 9, 11]. Other reports demonstrated that BMAA was detected in ALS/Parkinsonism dementia complex patients in Guam [26, 27]. These results can support the evidence that the cyanobacterial BMAA contributed to a risk factor of age-related neurodegenerations. In addition, Caller et al. [9] identified that BMAA was linked to the development of ALS and neurodegenerative diseases. However, very few studies have investigated potential linkages between HAB exposures and disease occurrences in Asia. Our previous study identified a significantly positive association between HABs intensity and non-alcoholic liver disease incidence rates in S. Korea [2]. There was an urgent need to determine the risks for other types of diseases related to HAB exposure in those bloom-affected areas. In this study, the associations between HAB intensity and human NDs, such as MND, AD, and PD, were identified with statistical analysis that factored in both time periods and distance. One notable finding in this study is that HABs have the most long-lasting effect on PD. Another highlight of this study is that distance of HAB can be a significant factor that impacts the occurrence of ND. These results imply that HABs can have long-term impacts on human health, especially those close to the bloom-prone major rivers in S. Korea.

Potential routes of exposure to cyanotoxin from HABs include ingestion of untreated/sub-optimally treated water; consuming fish, shellfish and crops; and inhaling aerosolized toxins. To examine the presence of airborne cyanobacteria and aerosolized cyanotoxin, a pilot study was conducted. Cyanobacteria were shown in a personal mask from a fisherman who works near the Nakdong River (Supplementary Fig. 1). In addition, BMAA and microcystin (MC) were determined from aerosol and water samples (Supplementary Table 2). The mean concentration of BMAA and MC and in the air sample was 6.8 ng/m^3^ and 16.1 ng/m^3^, respectively. Our results provide evidence that countries suffering from chronic HABs should make an effort to reduce HAB in their freshwater bodies as well as minimize exposure to HABs. For future epidemiological studies, samples from personal air samplers, nasal swabs, urine or blood samples for measurements of cyanotoxin exposures, including BMAA, can be helpful for understanding the extent of accurate cyanotoxin exposure and how the neurogenerative and other bloom-related diseases develop.

Although the roles of toxic HAB in neuropathy are still controversial, the statistical results of this study imply a significant correlation between HAB incidence and the occurrence of neurodegenerative diseases. Furthermore, our findings contribute to a greater understanding of toxic HAB with potential health risks because environmental alteration resulting from human impacts has increased HAB proliferation and persistence. Our study can provide awareness and warnings for people moving to areas closer to hotspots of bloom-area or coming into contact with the blooms as recreation, business, housing, etc. Finally, the modified statistic model with a grid-level population in this study can apply to other studies about determining correlations between potential health risks and other environmental contaminant exposures.

It should be noted that the findings from this study do not manifest a causal relationship between the HAB severity and human neurodegenerative diseases. However, this study provides statistical evidence that the HAB severity was a significant factor related to health risks, especially the occurrence of neurodegenerative diseases that have not been reported before.

The major limitation of this study is that chl-*a* values were used as an indicator for HAB intensity, instead of cyanotoxins because toxin data from the study sites and during the duration covered in this study were very much limited. Therefore, there is a possibility that using chl-*a* as a bloom indicator may over- or under-estimate potential health risks.

## Supplementary Information

**Additional file 1: Supplementary Table 1.** Results of GLM that examined the relationships between HABs and neurodegenerative diseases^α^ with different sizes of radius. **Supplementary Fig. 1.** Microscopic images (× 400) of cyanobacteria were captured from an outer layer of the personal mask from a fisherman working downstream of the Nakdong River (Daedong Dock (35.239992 (latitude), 128.996558 (longitude)). **Supplementary Table 2.** Concentrations of microcystins and BMAA from the air and water samples collected downstream of Nakdong River (Daedong Dock (35.239992 (latitude), 128.996558 (longitude)).

## Data Availability

The data of neurodegenerative diseases, chlorophyll-a, and gridded population used in the study can be accessed at https://github.com/timonpig/Korea-HAB-Neurodegerative-Disease.

## Abbreviations

AD: Alzheimer’s disease
ALS: Amyotrophic lateral sclerosis
BMAA: β-methylamino-L-alanine
GLM: Generalized linear model
GLMM: Generalized linear mixed model
HAB: Harmful algal bloom
MND: Motor neuron disease
ND: Neurodegenerative disease
PD: Parkinson’s disease
