# Supplementary material for: Relationship between freshwater harmful algal blooms and neurodegenerative disease incidence rates in South Korea
Source: Environ Health. 2022 Nov 26;21:116. doi: 10.1186/s12940-022-00935-y (PMC9700969; doi:10.1186/s12940-022-00935-y)
Supplement: Supplementary file 1 — Additional file 1: Supplementary Table 1. Results of GLM that examined the relationships between HABs and neurodegenerative diseasesα with different sizes of radius. Supplementary Fig. 1. Microscopic images (× 400) of cyanobacteria were captured from an outer layer of the personal mask from a fisherman working downstream of the Nakdong River (Daedong Dock (35.239992 (latitude), 128.996558 (longitude)). Supplementary Table 2. Concentrations of microcystins and BMAA from the air and water samples collected downstream of Nakdong River (Daedong Dock (35.239992 (latitude), 128.996558 (longitude)). [file 12940_2022_935_MOESM1_ESM.docx]

**Supplementary Material**

**Relationship between freshwater harmful algal blooms and neurodegenerative disease incidence rates in South Korea**

Seungjun Lee^a,§^, Boseung Choi^b,c,§^, Sung Jae Kim^d^, Jinnam Kim^e^, Dayun Kang^a^ and Jiyoung Lee^f,g,h^*

^a^ Department of Food Science and Nutrition, College of Fisheries Science, Pukyong National University, Busan, Republic of Korea

^b^ Division of Big Data Science Korea University, Sejong, Republic of Korea

^c^ Biomedical Mathematics Group, Institute for Basic Science, Daejeon, Republic of Korea

^d^ Department of Economics and Statistics, Korea University, Sejong, Republic of Korea

^e^ Department of Biology, Kyungsung University, Busan, Republic of Korea

^f^ College of Public Health, Division of Environmental Health Sciences, The Ohio State University, Columbus, OH, USA

^g^ Department of Food Science and Technology, The Ohio State University, Columbus, OH, USA

^h^ Infectious Diseases Institute, The Ohio State University, Columbus, OH, USA

^§^Equal contribution

*Corresponding author: Jiyoung Lee, [lee.3598@osu.edu](mailto:lee.3598@osu.edu)

406 Cunz Hall, 1841 Neil Avenue, Columbus, Ohio 43210, USA

**Supplementary Table 1**. Results of GLM that examined the relationships between HABs and neurodegenerative diseases^α^ with different sizes of radius.

| Senile  Disease | Radius | Parameter | Estimate | Standard  Error | Wald  Chi-Square | p |
| --- | --- | --- | --- | --- | --- | --- |
| MND | 3 km | Intercept | -12.1713 | 0.1453 | 7013.40 | <0.0001 |
|  |  | HABs | 0.0235 | 0.0721 | 0.11 | 0.7450 |
|  | 5 km | Intercept | -11.4391 | 0.1321 | 7501.99 | <0.0001 |
|  |  | HABs | 0.0948 | 0.0656 | 2.09 | 0.1486 |
| Alzheimer’s disease | 3 km | Intercept | -9.5607 | 0.1328 | 5183.42 | <0.0001 |
|  |  | HABs | -0.0384 | 0.0660 | 0.34 | 0.5604 |
|  | 5 km | Intercept | -8.8246 | 0.1197 | 5435.80 | <.0001 |
|  |  | HABs | 0.0328 | 0.0595 | 0.30 | 0.05816 |
| Parkinson’s disease | 3 km | Intercept | -8.5594 | 0.1221 | 4916.10 | <0.0001 |
|  |  | HABs | 0.0462 | 0.0607 | 0.58 | 0.4465 |
|  | 5 km | Intercept | -7.8336 | 0.1078 | 5281.68 | <0.0001 |
|  |  | HABs | 0.1185 | 0.0536 | 4.89 | 0.0271 |

MND: Motor-neuron disease

^a^ Based on the numbers of diseases in each grid’s adjusted population as defined in Eq. (2).

**A pilot study of airborne cyanobacteria and aerosolized BMAA and microcystin in Nakdong River, South Korea**

Aerosolization of toxic cyanobacteria/cyanotoxins can be a significant route of exposure to people during recreational activity and people who live or work near the bloom areas (Murby et al., 2016). A review paper reported adverse health outcomes associated with cyanotoxin inhalation (Plaas and Paerl, 2020). However, there has been no study of aerosolization of toxic cyanobacteria/cyanotoxin in South Korea. In this pilot study, we tested our hypotheses: 1) personal masks can be used as an easy-to-use screening tool for airborne cyanobacteria, and 2) cyanotoxins can be aerosolized in the area near the heavy bloom-affected water bodies.

The figure below shows cyanobacteria captured by a personal mask (average pore size, 0.4 µm; Supplementary Figure 1). It indicates a need for future studies to develop a better method to collect airborne cyanobacteria and monitor their levels, especially in the areas where frequent human and animal activities occur.

| 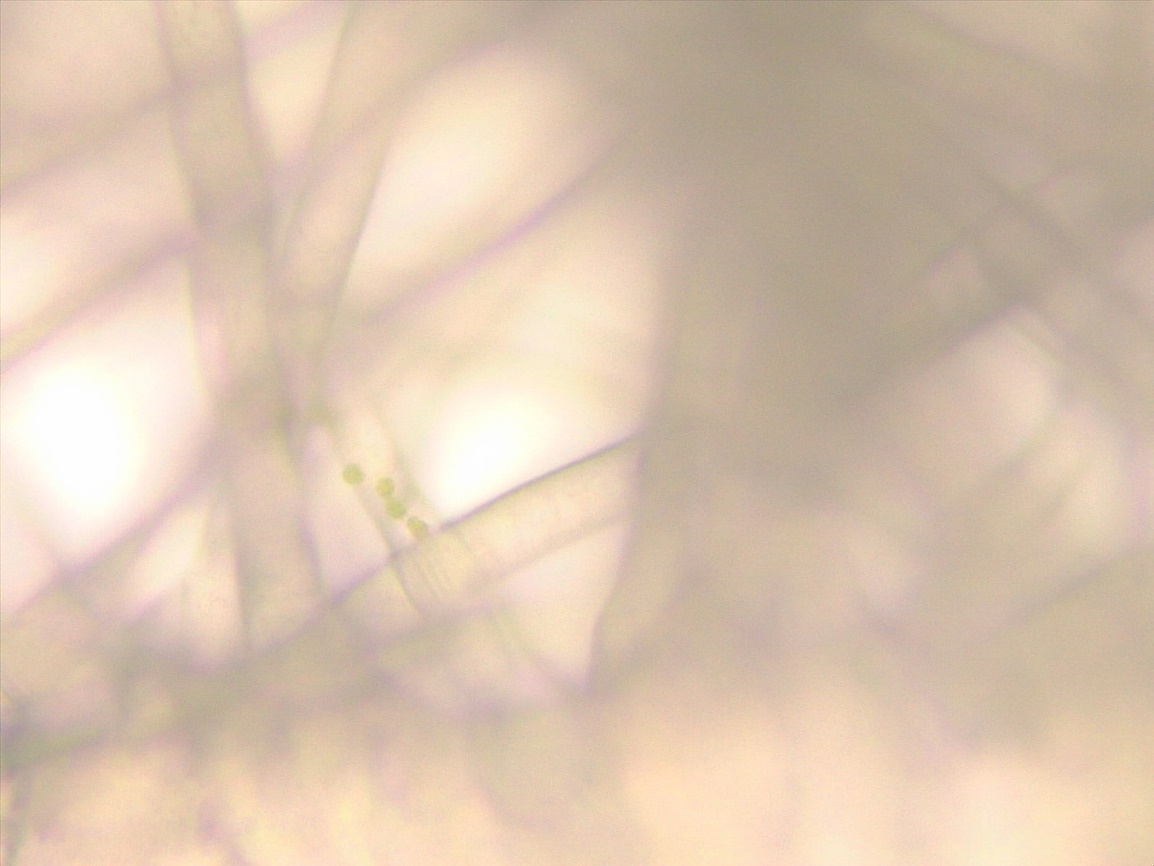 |
| --- |
| 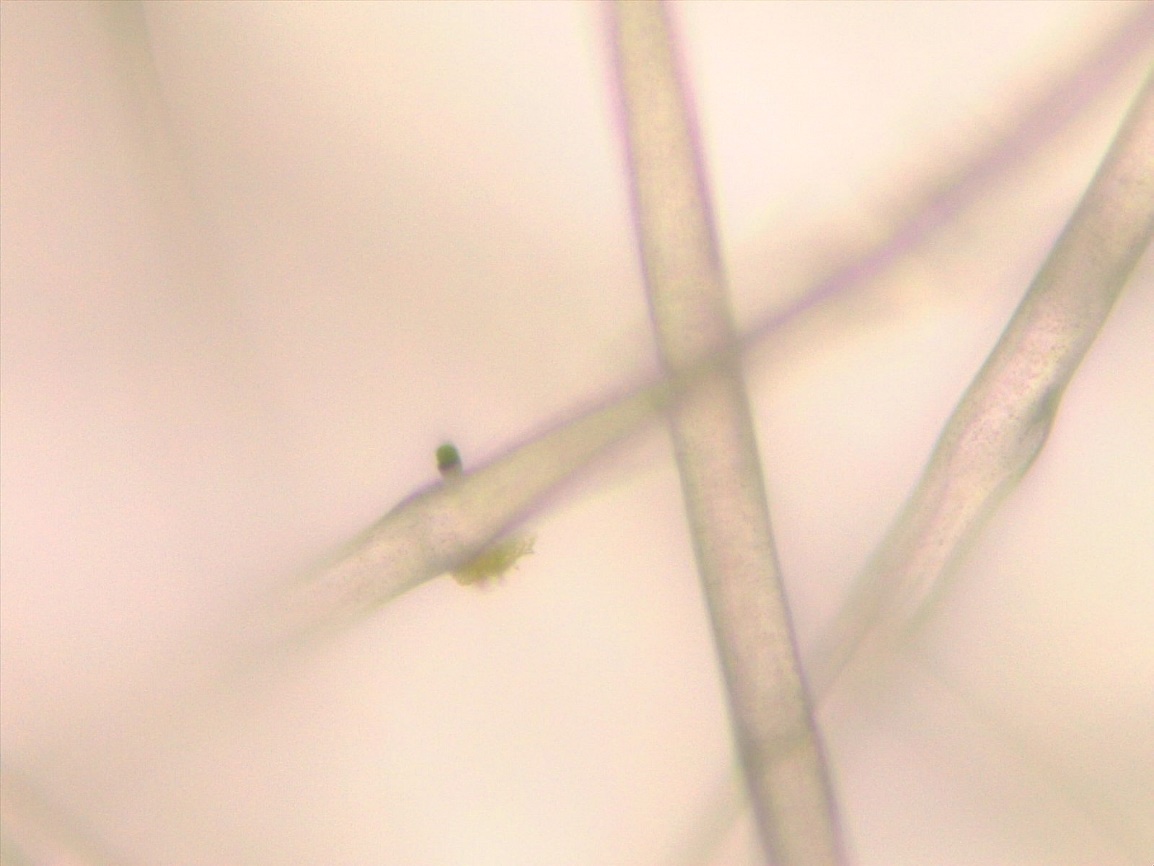 |
| **Supplementary Figure 1.** Microscopic images (×400) of cyanobacteria were captured from an outer layer of the personal mask from a fisherman working downstream of the Nakdong River (Daedong Dock (35.239992 (latitude), 128.996558 (longitude)) |

For the second hypothesis, as an initial trial, air samples were collected from the same location located downstream of the Nakdong River using air monitoring vacuum pumps (Air Pump SIP-32L, Sibata, South Korea) adjusted to a flow rate of 20 L/min (LPM) with a sterile membrane filter (mixed cellulose ester membrane, 37 mm, pore size 0.8µm, Sibata, South Korea). Air samples were collected for 4 hours (12:30 pm 4:30 pm) in the field during the bloom season. In addition, 100 mL of water sample was collected hourly (total volume, 400 mL). To measure cyanotoxins from the air samples, the cyanotoxin were extracted from the membrane filter following a previous study (Murby et al., 2016). Briefly, the membrane filter was mixed with 1 mL of distilled water, and then the sample was frozen and thawed three times with sonication (1 min) and vortexing (30 sec) between cycles. After concentrating by lyophilizing, two toxins were measured using enzyme-linked immunosorbent assays (ELISA): MC was measured with microcystins-ADDA SAES ELISA (Eurofins Abraxis, detection range of 0.05-5 µg/L) and BMAA was with BMAA ELISA (Eurofins Abraxis, detection range of 5-500 µg/L). To examine total microcystins (MC) concentrations in the water sample, the freeze-thaw lysis method was used (USEPA method 546). All experimental trials were repeated three times.

The mean concentration of MC and BMAA in the air sample was 6.8 ng/m^3^ and 16.1 ng/m^3^, respectively, while the mean concentration of MC and BMAA in the water sample was 64.2 µg/L and 8.0 µg/L, respectively (Supplementary Table 2).

**Supplementary Table 2**. Concentrations of microcystins and BMAA from the air and water samples collected downstream of Nakdong River (Daedong Dock (35.239992 (latitude), 128.996558 (longitude))

| Toxins | Sample type | Mean concentration |
| --- | --- | --- |
| Microcystis | Water | 64.2 µg/L |
|  | Air | 6.8 ng/m^3^ |
| BMAA | Water | 8.0 µg/L |
|  | Air | 16.1 ng/m^3^ |

As far as we know, this is the first study investigating the levels of aerosolized BMAA and microcystin and the presence of airborne cyanobacteria near the bloom area in South Korea. This pilot study on aerosolized cyanobacteria/cyanotoxin supports our hypotheses. The preliminary results warrant future research to develop better methods for detecting cyanotoxins from aerosol samples and investigate the inhalation exposure pathway as well as the ingestion pathway to protect public health from cyanotoxin risks. We speculate that the manifest symptoms from inhalation exposures can differ from the ingestion pathways.

**REFERENCES**

Murby, AL, Haney JF. Field and laboratory methods to monitor lake aerosols for cyanobacteria and microcystins. Aerobiologia, 2016; 32(3), 395-403.

Plaas HE, Paerl HW. Toxic cyanobacteria: a growing threat to water and air quality. Environmental science & technology, 2020; 55(1), 44-64.
